# (*E*)-3-(Oxolan-2-yl­idene)-1-phenyl­pyrrolidine-2,5-dione

**DOI:** 10.1107/S1600536814007193

**Published:** 2014-04-05

**Authors:** Ying Shao, Yong-An Xia, Zhu-Hong Wu, Xiao-Long Liu

**Affiliations:** a Key Laboratory of Fine Petrochemical Engineering, Changzhou University, Changzhou 213164, Jiangsu, People’s Republic of China

## Abstract

In the title compound, C_14_H_13_NO_3_, the dihedral angles between the central pyrrolidine ring and the pendant tetra­hydro­furan and phenyl rings are 5.34 (18) and 58.99 (17)°, respectively. The tetra­hydro­furan ring is almost planar (r.m.s. deviation = 0.008 Å). In the crystal, mol­ecules are linked by C—H⋯O inter­actions, generating a three-dimensional network.

## Related literature   

For synthetic background, see: Han *et al.* (2013 ▶); Sodhi *et al.* (2012 ▶).
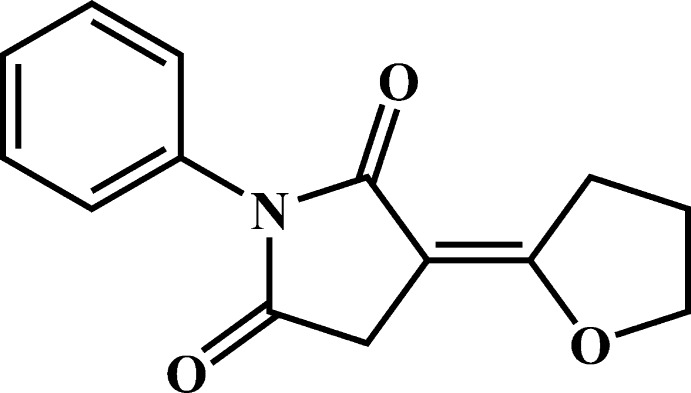


## Experimental   

### 

#### Crystal data  

C_14_H_13_NO_3_
*M* *_r_* = 243.25
Monoclinic, 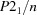

*a* = 8.144 (2) Å
*b* = 13.729 (4) Å
*c* = 11.160 (3) Å
β = 105.177 (8)°
*V* = 1204.2 (6) Å^3^
*Z* = 4
Mo *K*α radiation
μ = 0.10 mm^−1^
*T* = 296 K
0.30 × 0.28 × 0.25 mm

#### Data collection  

Rigaku Mercury CCD diffractometer
Absorption correction: multi-scan (*CrystalClear*; Rigaku, 2000 ▶) *T*_min_ = 0.736, *T*_max_ = 0.977
11234 measured reflections
2201 independent reflections
1893 reflections with *I* > 2σ(*I*)
*R*_int_ = 0.035

#### Refinement  

*R*[*F* ^2^ > 2σ(*F* ^2^)] = 0.075
*wR*(*F* ^2^) = 0.161
*S* = 1.08
2201 reflections
164 parameters
H-atom parameters constrained
Δρ_max_ = 0.23 e Å^−3^
Δρ_min_ = −0.21 e Å^−3^

### 

Data collection: *CrystalClear* (Rigaku, 2000 ▶); cell refinement: *CrystalClear*; data reduction: *CrystalStructure* (Rigaku, 2000 ▶); program(s) used to solve structure: *SHELXS97* (Sheldrick, 2008 ▶); program(s) used to refine structure: *SHELXL97* (Sheldrick, 2008 ▶); molecular graphics: *SHELXTL* (Sheldrick, 2008 ▶); software used to prepare material for publication: *SHELXTL*.

## Supplementary Material

Crystal structure: contains datablock(s) I, New_Global_Publ_Block. DOI: 10.1107/S1600536814007193/hb7212sup1.cif

Structure factors: contains datablock(s) I. DOI: 10.1107/S1600536814007193/hb7212Isup2.hkl

Supporting information file. DOI: 10.1107/S1600536814007193/hb7212Isup3.cdx

Supporting information file. DOI: 10.1107/S1600536814007193/hb7212Isup4.cml

CCDC reference: 994793

Additional supporting information:  crystallographic information; 3D view; checkCIF report

## Figures and Tables

**Table 1:** Hydrogen-bond geometry (Å, °)

*D*—H⋯*A*	*D*—H	H⋯*A*	*D*⋯*A*	*D*—H⋯*A*
C6—H6⋯O2^i^	0.93	2.59	3.487 (4)	161
C9—H9*A*⋯O1^ii^	0.97	2.50	3.403 (3)	154
C14—H14*A*⋯O2^iii^	0.97	2.50	3.376 (4)	150
C14—H14*B*⋯O2^iv^	0.97	2.51	3.384 (4)	149

Symmetry codes: (i) 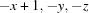; (ii) 
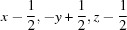
; (iii) 
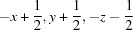
; (iv) 
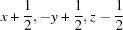
.

**Fig. 1.**
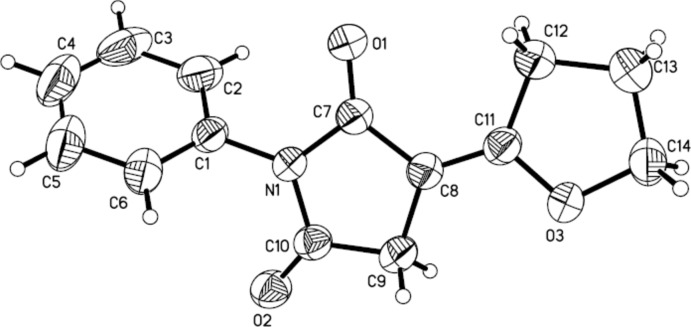
View of the title compound, showing 50% probability ellipsoids.

**Fig. 2.**
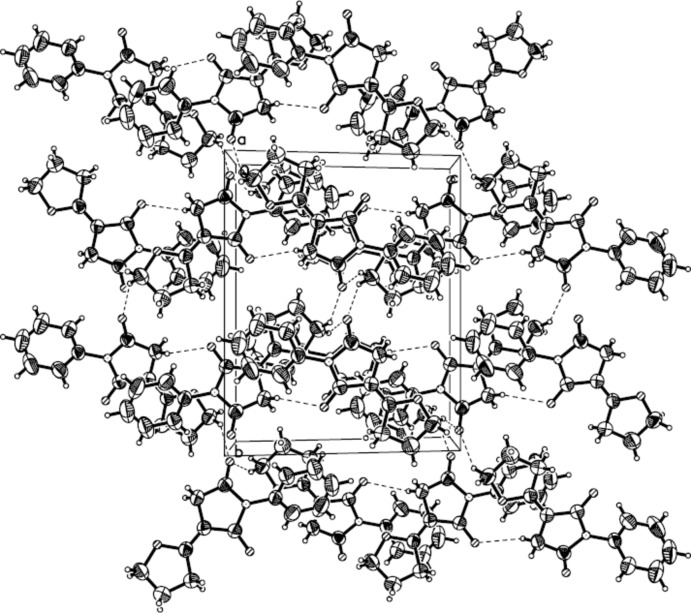
Perspective view of the packing of the title compound along a direction.

## supplementary crystallographic information

### 1. Comment

The area of allylic and benzylic oxidation with *tert*-butyl hydroperoxide (TBHP) in the presence of Co(acac)_2_ has been attracted more and more attention (Han, *et al.*, 2013; Sodhi, *et al.*, 2012). The title compound, C_14_H_13_NO_3_, was synthesized by Co(acac)_2_ catalyzed cross-dehydrogenative-coupling (CDC) between 1-phenyl-1*H*-pyrrole-2,5-dione and tetrahydrofuran in the presence of *t*-BuOOH as a oxidant in the air. In the molecule of the title compound (Fig. 1), the compound adopts an E conformation. All the non-H atoms of the pyrrolidine-2,5-dione and the tetrahydrofuran fragment, linked by carbon—carbon double bond, are nearly coplanar, with a maximum deviation of 0.056 (1) Å. While the dihedral angle between the benzene ring and the pyrrolidine-2,5-dione ring is 59.9 Å. In the crystal, C—H···O interactions link the molecules (Table 1).

### 2. Experimental

1-Phenyl-1*H*-pyrrole-2,5-dione(86.6 mg, 0.5 mmol), THF (0.5 ml, 7.0 mmol), cobalt(II) acetylacetonate (12.9 mg), 1,4-diazabicyclo[2.2.2]octane (70.1 mg, 0.6 mmol), TBHP (2.0 equiv, 70% aqueous solution 140 uL), 1.0 ml acetonitrile, 1.0 ml 1,4-dioxane were added to a tube under air. The reaction mixture was stirred at 60 *^o^*C for 4 h. Then the reaction mixture was quenched with saturated Na_2_SO_3_ solution_,_ extracted repeatedly with ethyl acetate, and dried over Na_2_SO_4_. It was then removal of the organic solvent in vacuum and followed by flash silica gel column chromatographic purification afforded product with petroleum/ ethyl acetate mixtures. Yield 40%. Colourless crystals were obtained by slow evaporation of ethyl acetate and CH_2_Cl_2_ mixed solvent.

### 3. Refinement

All the H atoms were placed in geometrically idealized positions and constrained to ride on their parent atoms, with C—H distances of 0.93–0.97 Å, and with *U*_iso_(H) = 1.2*U*_eq_(C).

### Crystal data

**Table:**

C_14_H_13_NO_3_	*F*(000) = 512
*M**_r_* = 243.25	*D*_x_ = 1.342 Mg m^−^^3^
Monoclinic, *P*2_1_/*n*	Mo *K*α radiation, λ = 0.71070 Å
Hall symbol: -P 2yn	Cell parameters from 4321 reflections
*a* = 8.144 (2) Å	θ = 3.2–25.3°
*b* = 13.729 (4) Å	µ = 0.10 mm^−^^1^
*c* = 11.160 (3) Å	*T* = 296 K
β = 105.177 (8)°	Block, colorless
*V* = 1204.2 (6) Å^3^	0.30 × 0.28 × 0.25 mm
*Z* = 4	

### Data collection

**Table:**

Rigaku Mercury CCD diffractometer	2201 independent reflections
Radiation source: fine-focus sealed tube	1893 reflections with *I* > 2σ(*I*)
Graphite monochromator	*R*_int_ = 0.035
Detector resolution: 7.31 pixels mm^-1^	θ_max_ = 25.3°, θ_min_ = 3.2°
ω scans	*h* = −9→9
Absorption correction: multi-scan (*CrystalClear*; Rigaku, 2000)	*k* = −16→15
*T*_min_ = 0.736, *T*_max_ = 0.977	*l* = −13→13
11234 measured reflections	

### Refinement

**Table:**

Refinement on *F*^2^	Primary atom site location: structure-invariant direct methods
Least-squares matrix: full	Secondary atom site location: difference Fourier map
*R*[*F*^2^ > 2σ(*F*^2^)] = 0.075	Hydrogen site location: inferred from neighbouring sites
*wR*(*F*^2^) = 0.161	H-atom parameters constrained
*S* = 1.08	*w* = 1/[σ^2^(*F*_o_^2^) + (0.0555*P*)^2^ + 0.8273*P*] where *P* = (*F*_o_^2^ + 2*F*_c_^2^)/3
2201 reflections	(Δ/σ)_max_ < 0.001
164 parameters	Δρ_max_ = 0.23 e Å^−^^3^
0 restraints	Δρ_min_ = −0.21 e Å^−^^3^

### Special details

**Table:**

Geometry. All e.s.d.'s (except the e.s.d. in the dihedral angle between two l.s. planes) are estimated using the full covariance matrix. The cell e.s.d.'s are taken into account individually in the estimation of e.s.d.'s in distances, angles and torsion angles; correlations between e.s.d.'s in cell parameters are only used when they are defined by crystal symmetry. An approximate (isotropic) treatment of cell e.s.d.'s is used for estimating e.s.d.'s involving l.s. planes.
Refinement. Refinement of *F*^2^ against ALL reflections. The weighted *R*-factor *wR* and goodness of fit *S* are based on *F*^2^, conventional *R*-factors *R* are based on *F*, with *F* set to zero for negative *F*^2^. The threshold expression of *F*^2^ > σ(*F*^2^) is used only for calculating *R*-factors(gt) *etc*. and is not relevant to the choice of reflections for refinement. *R*-factors based on *F*^2^ are statistically about twice as large as those based on *F*, and *R*- factors based on ALL data will be even larger.

### Fractional atomic coordinates and isotropic or equivalent isotropic displacement parameters (Å^2^)

**Table:**

	*x*	*y*	*z*	*U*_iso_*/*U*_eq_	
O1	0.5949 (3)	0.34863 (14)	0.08693 (17)	0.0660 (6)	
O2	0.3330 (3)	0.05454 (15)	−0.01104 (17)	0.0632 (6)	
O3	0.4751 (3)	0.32363 (14)	−0.31330 (16)	0.0612 (6)	
N1	0.4759 (3)	0.19466 (15)	0.06629 (18)	0.0505 (6)	
C1	0.5003 (3)	0.1742 (2)	0.1954 (2)	0.0526 (7)	
C2	0.4297 (4)	0.2340 (2)	0.2671 (3)	0.0720 (9)	
H2	0.3637	0.2873	0.2323	0.086*	
C3	0.4596 (6)	0.2129 (3)	0.3938 (3)	0.0938 (13)	
H3	0.4153	0.2531	0.4447	0.113*	
C4	0.5547 (6)	0.1324 (4)	0.4428 (3)	0.0986 (14)	
H4	0.5739	0.1185	0.5269	0.118*	
C5	0.6209 (5)	0.0727 (3)	0.3697 (3)	0.0866 (11)	
H5	0.6835	0.0180	0.4035	0.104*	
C6	0.5947 (4)	0.0937 (2)	0.2458 (3)	0.0639 (8)	
H6	0.6406	0.0536	0.1957	0.077*	
C7	0.5310 (3)	0.28121 (19)	0.0200 (2)	0.0514 (6)	
C8	0.4917 (3)	0.27047 (19)	−0.1134 (2)	0.0507 (6)	
C9	0.4145 (4)	0.1720 (2)	−0.1479 (2)	0.0571 (7)	
H9A	0.3038	0.1775	−0.2069	0.069*	
H9B	0.4875	0.1319	−0.1838	0.069*	
C10	0.3991 (3)	0.1303 (2)	−0.0279 (2)	0.0503 (6)	
C11	0.5168 (3)	0.34047 (19)	−0.1899 (2)	0.0508 (7)	
C12	0.5857 (4)	0.4404 (2)	−0.1610 (3)	0.0603 (7)	
H12A	0.7015	0.4385	−0.1089	0.072*	
H12B	0.5161	0.4776	−0.1189	0.072*	
C13	0.5797 (6)	0.4839 (3)	−0.2852 (3)	0.0956 (13)	
H13A	0.5089	0.5418	−0.2989	0.115*	
H13B	0.6932	0.5019	−0.2895	0.115*	
C14	0.5080 (4)	0.4095 (2)	−0.3792 (3)	0.0679 (8)	
H14A	0.4032	0.4329	−0.4351	0.081*	
H14B	0.5878	0.3943	−0.4276	0.081*	

### Atomic displacement parameters (Å^2^)

**Table:**

	*U*^11^	*U*^22^	*U*^33^	*U*^12^	*U*^13^	*U*^23^
O1	0.0912 (15)	0.0522 (11)	0.0478 (11)	−0.0092 (11)	0.0059 (10)	−0.0045 (9)
O2	0.0689 (13)	0.0623 (12)	0.0580 (11)	−0.0180 (10)	0.0161 (9)	−0.0065 (10)
O3	0.0833 (14)	0.0570 (11)	0.0427 (10)	−0.0044 (10)	0.0154 (9)	0.0014 (9)
N1	0.0628 (14)	0.0465 (12)	0.0390 (11)	−0.0043 (10)	0.0080 (9)	−0.0018 (9)
C1	0.0590 (16)	0.0550 (16)	0.0420 (14)	−0.0120 (13)	0.0103 (12)	−0.0030 (12)
C2	0.086 (2)	0.068 (2)	0.0651 (19)	−0.0158 (17)	0.0259 (17)	−0.0166 (16)
C3	0.120 (3)	0.108 (3)	0.066 (2)	−0.048 (3)	0.046 (2)	−0.038 (2)
C4	0.114 (3)	0.128 (4)	0.048 (2)	−0.053 (3)	0.010 (2)	0.008 (2)
C5	0.084 (2)	0.112 (3)	0.056 (2)	−0.020 (2)	0.0052 (17)	0.022 (2)
C6	0.0620 (18)	0.073 (2)	0.0542 (17)	−0.0030 (15)	0.0099 (13)	0.0091 (14)
C7	0.0576 (16)	0.0468 (15)	0.0464 (14)	0.0016 (12)	0.0077 (12)	−0.0012 (12)
C8	0.0559 (16)	0.0484 (14)	0.0445 (14)	0.0023 (12)	0.0074 (11)	−0.0001 (12)
C9	0.0667 (18)	0.0587 (17)	0.0431 (14)	−0.0069 (14)	0.0092 (12)	−0.0059 (12)
C10	0.0478 (15)	0.0512 (15)	0.0496 (15)	0.0000 (12)	0.0085 (11)	−0.0050 (12)
C11	0.0532 (15)	0.0532 (15)	0.0435 (14)	0.0046 (12)	0.0080 (11)	−0.0025 (12)
C12	0.0725 (19)	0.0493 (15)	0.0572 (16)	0.0014 (14)	0.0136 (14)	−0.0015 (13)
C13	0.153 (4)	0.067 (2)	0.069 (2)	−0.030 (2)	0.032 (2)	0.0000 (18)
C14	0.086 (2)	0.0648 (19)	0.0566 (17)	0.0054 (16)	0.0264 (16)	0.0122 (15)

### Geometric parameters (Å, º)

**Table:**

O1—C7	1.217 (3)	C6—H6	0.9300
O2—C10	1.208 (3)	C7—C8	1.447 (4)
O3—C11	1.349 (3)	C8—C11	1.336 (4)
O3—C14	1.451 (3)	C8—C9	1.498 (4)
N1—C10	1.390 (3)	C9—C10	1.492 (4)
N1—C7	1.414 (3)	C9—H9A	0.9700
N1—C1	1.430 (3)	C9—H9B	0.9700
C1—C2	1.372 (4)	C11—C12	1.486 (4)
C1—C6	1.379 (4)	C12—C13	1.498 (4)
C2—C3	1.402 (5)	C12—H12A	0.9700
C2—H2	0.9300	C12—H12B	0.9700
C3—C4	1.378 (6)	C13—C14	1.472 (4)
C3—H3	0.9300	C13—H13A	0.9700
C4—C5	1.364 (6)	C13—H13B	0.9700
C4—H4	0.9300	C14—H14A	0.9700
C5—C6	1.374 (4)	C14—H14B	0.9700
C5—H5	0.9300		
			
C11—O3—C14	110.3 (2)	C10—C9—H9A	110.9
C10—N1—C7	112.4 (2)	C8—C9—H9A	110.9
C10—N1—C1	123.6 (2)	C10—C9—H9B	110.9
C7—N1—C1	124.0 (2)	C8—C9—H9B	110.9
C2—C1—C6	121.0 (3)	H9A—C9—H9B	108.9
C2—C1—N1	120.0 (3)	O2—C10—N1	124.1 (2)
C6—C1—N1	118.9 (3)	O2—C10—C9	128.0 (2)
C1—C2—C3	118.4 (4)	N1—C10—C9	107.9 (2)
C1—C2—H2	120.8	C8—C11—O3	119.3 (2)
C3—C2—H2	120.8	C8—C11—C12	129.6 (2)
C4—C3—C2	119.8 (4)	O3—C11—C12	111.1 (2)
C4—C3—H3	120.1	C11—C12—C13	104.4 (2)
C2—C3—H3	120.1	C11—C12—H12A	110.9
C5—C4—C3	121.0 (3)	C13—C12—H12A	110.9
C5—C4—H4	119.5	C11—C12—H12B	110.9
C3—C4—H4	119.5	C13—C12—H12B	110.9
C4—C5—C6	119.6 (4)	H12A—C12—H12B	108.9
C4—C5—H5	120.2	C14—C13—C12	107.1 (3)
C6—C5—H5	120.2	C14—C13—H13A	110.3
C5—C6—C1	120.1 (3)	C12—C13—H13A	110.3
C5—C6—H6	119.9	C14—C13—H13B	110.3
C1—C6—H6	119.9	C12—C13—H13B	110.3
O1—C7—N1	122.7 (2)	H13A—C13—H13B	108.6
O1—C7—C8	130.7 (3)	O3—C14—C13	107.1 (2)
N1—C7—C8	106.5 (2)	O3—C14—H14A	110.3
C11—C8—C7	123.7 (2)	C13—C14—H14A	110.3
C11—C8—C9	127.5 (2)	O3—C14—H14B	110.3
C7—C8—C9	108.7 (2)	C13—C14—H14B	110.3
C10—C9—C8	104.1 (2)	H14A—C14—H14B	108.6
			
C10—N1—C1—C2	−120.8 (3)	C11—C8—C9—C10	−173.3 (3)
C7—N1—C1—C2	60.9 (4)	C7—C8—C9—C10	4.4 (3)
C10—N1—C1—C6	58.9 (4)	C7—N1—C10—O2	−175.2 (3)
C7—N1—C1—C6	−119.3 (3)	C1—N1—C10—O2	6.4 (4)
C6—C1—C2—C3	1.7 (4)	C7—N1—C10—C9	4.8 (3)
N1—C1—C2—C3	−178.6 (3)	C1—N1—C10—C9	−173.6 (2)
C1—C2—C3—C4	−1.4 (5)	C8—C9—C10—O2	174.5 (3)
C2—C3—C4—C5	0.1 (6)	C8—C9—C10—N1	−5.5 (3)
C3—C4—C5—C6	0.9 (6)	C7—C8—C11—O3	−179.6 (2)
C4—C5—C6—C1	−0.7 (5)	C9—C8—C11—O3	−2.2 (4)
C2—C1—C6—C5	−0.6 (4)	C7—C8—C11—C12	−0.3 (5)
N1—C1—C6—C5	179.6 (3)	C9—C8—C11—C12	177.1 (3)
C10—N1—C7—O1	176.9 (3)	C14—O3—C11—C8	178.4 (3)
C1—N1—C7—O1	−4.7 (4)	C14—O3—C11—C12	−1.0 (3)
C10—N1—C7—C8	−1.9 (3)	C8—C11—C12—C13	−179.0 (3)
C1—N1—C7—C8	176.5 (2)	O3—C11—C12—C13	0.3 (4)
O1—C7—C8—C11	−2.6 (5)	C11—C12—C13—C14	0.5 (4)
N1—C7—C8—C11	176.1 (2)	C11—O3—C14—C13	1.3 (4)
O1—C7—C8—C9	179.6 (3)	C12—C13—C14—O3	−1.0 (4)
N1—C7—C8—C9	−1.7 (3)		

### Hydrogen-bond geometry (Å, º)

**Table:**

*D*—H···*A*	*D*—H	H···*A*	*D*···*A*	*D*—H···*A*
C6—H6···O2^i^	0.93	2.59	3.487 (4)	161
C9—H9*A*···O1^ii^	0.97	2.50	3.403 (3)	154
C14—H14*A*···O2^iii^	0.97	2.50	3.376 (4)	150
C14—H14*B*···O2^iv^	0.97	2.51	3.384 (4)	149

Symmetry codes: (i) −*x*+1, −*y*, −*z*; (ii) *x*−1/2, −*y*+1/2, *z*−1/2; (iii) −*x*+1/2, *y*+1/2, −*z*−1/2; (iv) *x*+1/2, −*y*+1/2, *z*−1/2.
